# Sphingomyelin nanosystems loaded with uroguanylin and etoposide for treating metastatic colorectal cancer

**DOI:** 10.1038/s41598-021-96578-z

**Published:** 2021-08-26

**Authors:** Belén L. Bouzo, Saínza Lores, Raneem Jatal, Sandra Alijas, María José Alonso, Inmaculada Conejos-Sánchez, María de la Fuente

**Affiliations:** 1 Nano-Oncology and Translational Therapeutics Unit, Health Research Institute of Santiago de Compostela (IDIS), SERGAS, CIBERONC, 15706 Santiago de Compostela, Spain; 2 Centre for Research in Molecular Medicine and Chronic Diseases (CIMUS), University of Santiago de Compostela (USC), Av. Barcelona s/n Campus Vida, 15706 Santiago de Compostela, Spain; 3 Universidade de Santiago de Compostela (USC), 15782 Santiago de Compostela, Spain; 4 Health Research Institute of Santiago de Compostela (IDIS), 15706 Santiago de Compostela, Spain; 5 Faculty of Pharmacy, University of Santiago de Compostela, 15705 Santiago de Compostela, Spain

**Keywords:** Molecular medicine, Cancer, Cancer therapy, Nanomedicine, Drug delivery, Chemical modification

## Abstract

Colorectal cancer is the third most frequently diagnosed cancer malignancy and the second leading cause of cancer-related deaths worldwide. Therefore, it is of utmost importance to provide new therapeutic options that can improve survival. Sphingomyelin nanosystems (SNs) are a promising type of nanocarriers with potential for association of different types of drugs and, thus, for the development of combination treatments. In this work we propose the chemical modification of uroguanylin, a natural ligand for the Guanylyl Cyclase (GCC) receptor, expressed in metastatic colorectal cancer tumors, to favour its anchoring to SNs (UroGm-SNs). The anti-cancer drug etoposide (Etp) was additionally encapsulated for the development of a combination strategy (UroGm-Etp-SNs). Results from in vitro studies showed that UroGm-Etp-SNs can interact with colorectal cancer cells that express the GCC receptor and mediate an antiproliferative response, which is more remarkable for the drugs in combination. The potential of UroGm-Etp-SNs to treat metastatic colorectal cancer cells was complemented with an in vivo experiment in a xenograft mice model.

According to the World Health Organization (WHO), cancer causes more deaths than all heart diseases or strokes^1^. Colorectal cancer (CRC) is the third most frequently diagnosed cancer malignancy and the second leading cause of cancer-related deaths in the world, causing approximately 10% of deaths with an increase of over 20 million new cancer cases expected annually by 2025^2^. Moreover, the presence of local or distant metastasis remains the leading cause of death among cancer patients, with an overall mortality above 50%. These facts emphasize an unmet clinical need for the effective targeting of colorectal cancer metastasis^3^. Guanylyl Cyclase C receptor (GCC) is expressed at the apical membrane of enterocytes from duodenum to distal rectum and also by primary and metastatic colorectal cancer cells, however, it is not expressed in healthy extraintestinal tissue such as liver and lungs, where colorectal cancer cells usually metastasize^4–6^, being therefore a promising target to treat metastasis. GCC is activated upon binding to the paracrine hormones Guanylin (Gn) and Uroguanylin (UroG) as well as to the enterotoxigenic *Escherichia coli* heat stable enterotoxin (ST)^7^. GCC-paracrine hormones axis is considered a key regulator of several cellular processes such as differentiation, apoptosis, proliferation and migration^7–11^. With respect to its use in therapy, several studies performed in T84, HT29 and CaCo2 colorectal cancer cell lines showed that Gn, UroG and the ST enterotoxin can indeed inhibit cell proliferation based on GCC activation^5,12^. This clinical evidence prompted several GCC agonists already approved by the FDA (Linaclotide, Plecanatide and Dolcanatide) as potential candidates for oral cancer chemoprevention^13^. In line with these discoveries, some authors have reported the development of radiotracers, based on chemical modification of the endogenous agonists (UroG, Gn and ST) to target GCC for PET and SPECT molecular diagnosis^14–16^. To the best of our knowledge, the development of nanosystems modified with UroG to target GCC has not been reported to date, being this, one of the subjects of this study. The use of nanosystems for the development of anticancer therapeutics has several advantages, such as an increase in the therapeutic effectiveness, diminishing the administered dose, and/or decreasing the secondary effects by means of targeting strategies (active/passive) that increase drug accumulation into the tumors^17^. On the other hand, nanosystems offer the possibility to include a variety of anticancer drugs in a single carrier for the development of combination therapies^18^. Therefore, we proposed here the co-encapsulation of UroG with etoposide, an anticancer agent commonly used in clinics, that has proved to be efficient in cancer treatment mostly in combination with other drugs such as 5-fluorouracil, paclitaxel or doxorubicin^19–21^. Our group has recently developed a novel formulation, Sphingomyelin Nanosystems (SNs), and showed the potential of this biocompatible formulation for drug delivery and diagnosis applications^22–25^. We have proved that peptide modification with hydrophobic fatty acids improves their association efficiency to SNs^23^. Additionally, SNs are promising candidates for the development of combinatory therapies, since they can accommodate different therapeutic molecules, in terms of activity and physicochemical properties, very efficiently. Based on our previous results, and on the medical need of developing novel therapeutic approaches specifically designed to treat metastatic colorectal cancer cells, we propose here the development of a combination therapy by loading a UroG derivative (UroGm) upon conjugation to a PEG-lipid moiety and a conventional chemotherapeutic drug, etoposide, into SNs, for treating metastatic colorectal cancer cells expressing GCC. The potential of this combination therapy for treating metastatic colorectal cancer cells was investigated in vitro and confirmed in an experimental mice model.

## Results and discussion

Nanosystems intended for cancer treatment have been mostly designed relying on the intrinsic capacity of these small particles to enhance their circulation time and eventually undergo passive accumulation into the tumor tissues due to the enhanced permeability and retention effect (EPR effect)^26^. Recent studies highlighted the need for targeted therapies that could enhance the accumulation of nanoparticles in the tumor^27^. By means of active targeting, nanoparticles can theoretically achieve higher levels of drug concentration in tumour tissues via receptor-mediated endocytosis^17^. In fact, the use of targeting ligands has been shown to reduce side effects and improve the therapeutic output^28^. Typically, well-known receptors involved in tumour progression, such as HER2, folate receptor, CD44, and EGFR, have been exploited for that purpose^29^. One of the problems associated to these strategies relies on the fact that most of these receptors are non-specific for cancer cells but ubiquitously expressed in the body. Therefore, the identification of selective markers on tumor cells becomes critical to enable selectivity for tumor tissue over normal cells^30^. Considering this, we focus our attention on UroG, a peptide that naturally binds the Guanylyl Cyclase C (GCC) receptor, a very promising target that is expressed at the apical membrane of enterocytes, and by primary and metastatic colorectal cancer cells, but not by healthy extraintestinal tissue^4–6,9^.

### Synthesis and characterization of an UroG derivative (UroGm)

**Figure 1:**
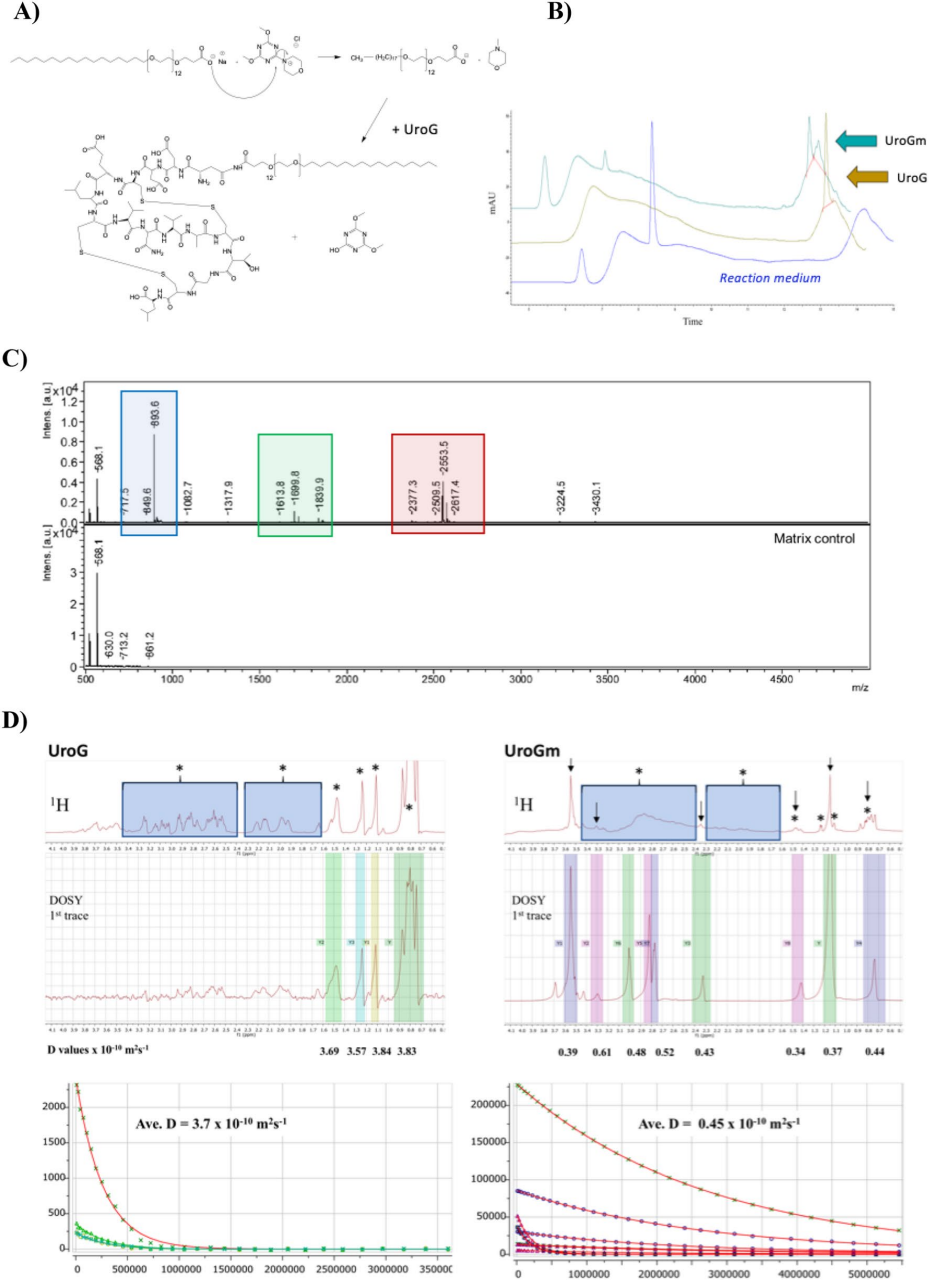
(**A**) Schematic representation of the chemical synthesis of the UroG-PEG_12_-C_18_ conjugate (UroGm) through DMTMM-carboxyl activation. (**B**) HPLC chromatograms for: (i) the reaction medium, COOH-PEG_12_-C_18_ and DMTMM·Cl dissolved in HEPES 150 mM (blue line), (ii) the standard unmodified peptide UroG (yellow line) and (iii) the purified compounds UroG + UroGm conjugate (turquoise line). (**C**) MALDI-TOF signals of COOH-PEG_12_-C_18_ (blue area), UroG unmodified peptide (green area) and purified conjugate UroGm (red area). (**D**) DOSY analysis of the parent peptide UroG (left) and its conjugate UroGm (right) (* = UroG signals, ↓ = PEG_12_-C_18_ signals). MestreNova software v11.0 (Mestrelab Research Inc., Spain).

In order to develop nanosystems that incorporate UroG, and considering previous reports from our research group proving that SNs can efficiently associate amphiphilic peptides^23^, first experiments were conducted to the preparation of an amphiphilic derivative of UroG. For this modification, we selected a poly(ethylene glycol) (PEG) derivative with a hydrophobic stearic acid chain (C_18_). PEGylated lipids are vastly used in nanoformulations as surface stabilizers and for improving the circulation half-life^31^. In the last decade, the bifunctionality of PEG has allowed further exploitation for the conjugation of bioactive molecules such as antibodies or peptides through a great variety of linkers, constructing cell-specific targeting nanocarriers^32,33^. This strategy will also allow promoting a successful insertion/anchoring of the peptide in SNs, while exposing UroG linked to the PEG section to the outer part, thus making it accessible for receptor recognition. The primary amino group of UroG was linked to the terminal carboxylic group of the amphiphilic surfactant COOH-PEG_12_-C_18_. The selection of an amide bond as the linkage functional group was based on its simplicity and non-immunogenicity, and was consistent with reported studies considering the importance of the carboxy-terminal conserved domain (cysteine domain) for the proper folding and bioactivity of the hormone^15,34^. Besides, the amide group provides a stable union between the peptide and the PEGylated lipid, avoiding a prompt release of the targeting moiety from the nanosystem under multiple biological scenarios^35^. Activation of the carboxyl group through DMTMM is a simple reaction where DMTMM chloride generates the activated ester releasing 4-methylmorpholine in the first step. An amide bond is then formed between the activated ester and the amine present (Fig. 1A). UroG reaction progress was monitored by HPLC, evidenced by a decreasing intensity of the original peptide peak along reaction time and its shifting from t_R_(UroG) = 13.8 min to t_R_(UroGm) = 14 min (Fig. 1B). Once the purification of the product was done, HPLC analysis indicated a 75.45% conjugation yield. In order to certify the conjugation and the identity of the conjugate, MALDI-TOF analyses were subsequently carried out (Fig. 1C). These data provided the molecular weight (MW) of the conjugate, approximately 2560 Da, which corresponds to the formation of a 1:1 conjugate. Next, this data was further corroborated by NMR analysis through: ^1^H-NMR (Figure S1A) and TOCSY (Figure S1B) experiments.

The diffusion coefficients (D) of the single reagents (UroG and PEG_12_-C_18_) and the reaction product (UroG-PEG_12_-C_18_ conjugate, UroGm) were also determined by diffusion NMR (DOSY-NMR) experiments (Fig. 1D). This parameter is directly related with many intrinsic properties of the molecule such as MW, size, shape and charge^36,37^. In the present study, a lower D value of the conjugate was obtained in comparison with non-modified peptide (D_UroG_ = 3.7 × 10^–10^ m^2^ s^−1^, D_UroGm_ = 0.45 × 10^–10^ m^2^ s^−1^, D_C18-PEG12_ = 0.46 × 10^–10^ m^2^ s^−1^) as expected since lower diffusion coefficients correspond to higher MW species. Although it was not possible to calculate the coefficient through the peptide peaks in the UroGm sample, due to a deficient signal-to-noise ratio, the PEG-lipid signals were highly robust to allow the calculation of the coefficient in a consistent manner. Finally, TOCSY analysis proved the presence of UroG in the conjugate sample (Figure S2). Conjugation is also proved due to the observation of broader peaks in the ^1^H-NMR spectra, a well-established characteristic after polymer conjugation, as well as the shifting of NH-signals of the UroGm in comparison with the pure peptide. Overall, the results of the three characterization techniques led us to conclude that conjugation prompted a structurally well-defined peptide-PEG-lipid (UroG-PEG_12_-C_18_), certifying that UroG peptide was successfully conjugated through an amide bond.

**Figure 2:**
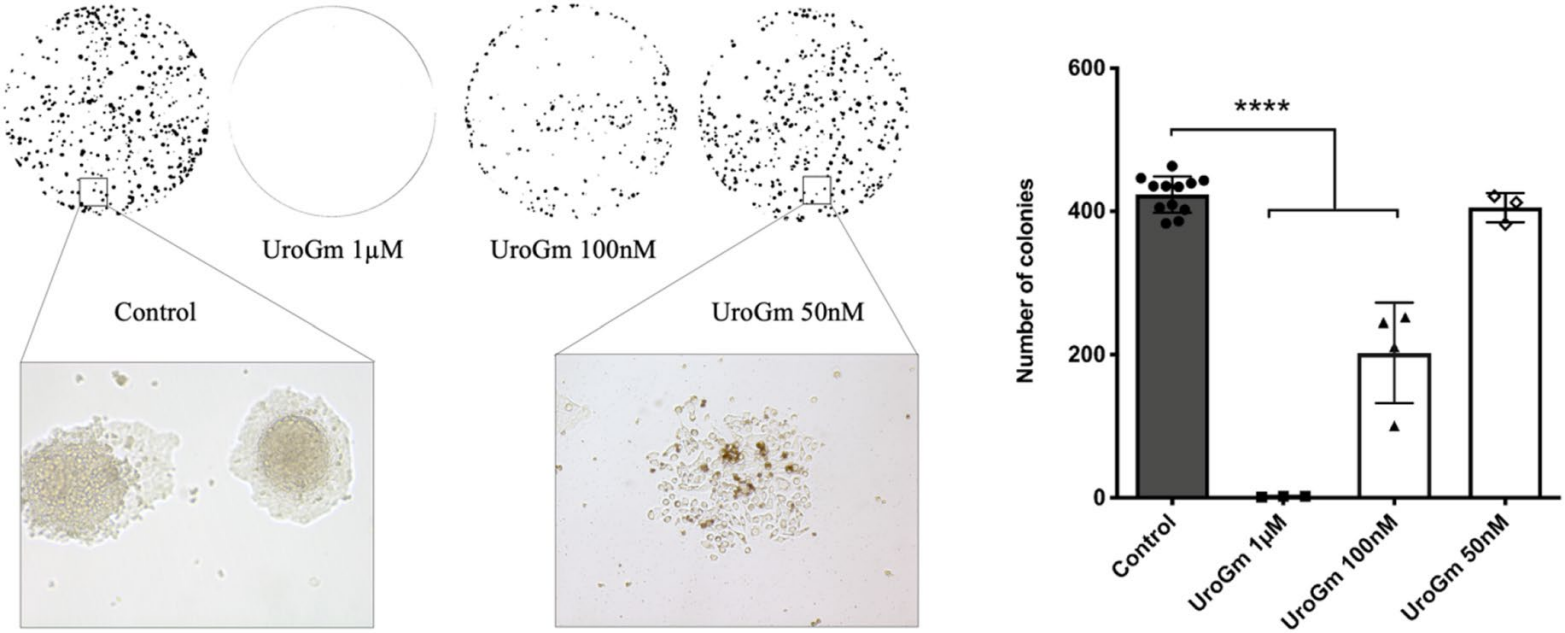
Evaluation of cell proliferation activity by colony forming assay (CFA) of SW620 cells treated with increasing UroGm concentrations. Left, image of macroscopic colonies photographed from P12 well plates and cellular detail of control and UroGm at 50 nM. Right, graphical representation. *P* value ****p < 0.0001.

After having proved that we successfully prepared a UroGm conjugate, we aimed to determine if the biological effect of UroG was preserved. For this, a tumor colony forming assay (CFA) was used to quantitatively evaluate the capacity of the molecule to impede the ability of a single cell to grow into a colony through clonal expansion^38,39^. This experiment was carried out in a metastatic colorectal cancer cell line that constitutively expresses the GCC receptor, SW620^40^ (expression level confirmed by immunofluorescence Figure S3). After the addition of increasing concentrations of UroGm in solution, from 50 nM to 1 µM, it was observed that the number of colonies significantly decreased following a concentration dependent pattern (Fig. 2). Our results prove that the biological function of the hormone remains intact despite the chemical modification, preserving the ability to target the GCC receptor and decrease cell tumorigenicity as a result of the activation of the cGMP-AKT axis^41,42^ for concentrations in the same order of magnitude as previously reported by other authors^5,12,43,44^.

### Association of UroGm to SNs

The selection of SNs as a delivery platform was based on previous results by our group, showing the advantages of this technology in drug delivery and molecular imaging applications^23–25^. Composed only by two natural lipids, SNs are biodegradable and biocompatible, can be prepared following a spontaneous emulsification low-energy manufacturing technique, are easily scalable, and have a long shelf-life, altogether possessing exceptional characteristics for clinical translation. Additionally, we have previously demonstrated, in silico and in wet experiments, that peptides (as is the case of UroG), could be efficiently associated to SNs when they are converted into amphiphiles bearing a hydrophobic tail^23^. Therefore the inclusion of the targeting molecule (UroGm) into the nanosystem was expected to happen through the C_18_ lipophilic segment of the conjugate. In that way, the hydrophilic targeting moiety would be oriented towards the external aqueous phase. UroGm was incorporated into the aqueous phase, followed by the addition of the lipids dissolved in ethanol. The physicochemical properties of the obtained nanosystems were exhaustively studied using a wide panel of analytical techniques. All nanosystems showed a nanometric size below 150 nm and a negative surface charge (Table 1). A decrease in the mean particle size was observed for the decorated UroGm-SNs (131 ± 12 nm) with respect to plain SNs (149 ± 10 nm). This variation could be associated with the ability of the PEG_12_-C_18_ to act as a capping agent due to the O–H bond present at the end of the molecule, which raises the hydrophilic character of the UroGm-SNs. The conjugated peptide could then behave as a new surfactant molecule due to its amphiphilic character^45^. Variation in charge towards more negative values was also observed indicating an efficient association of the negative conjugate (UroGm) to the nanosystem surface. Monodisperse populations were obtained for both SNs and UroGm-SNs, according to the homogeneity values, polydispersity index ≤ 0.2 and SPAN value ≤ 1.

**Table 1:** Physicochemical characterization of SNs and UroGm-SNs.

Formulation	Zetasizer (DLS and LDA)			Nanosight (NTA)					
	Size (nm)	PdI	ZP (mV)	Size (nm)	D_10_	D_50_	D_90_	SPAN	Conc. (particles/mL)
SNs	149 ± 10	0.2	− 23 ± 5	151 ± 3	107 ± 1	139 ± 3	208 ± 9	0.73	4.9 × 10^11^ ± 3.7 × 10^10^
UroGm-SNs	131 ± 12	0.2	− 44 ± 4	110 ± 2	72 ± 1	95 ± 1	152 ± 4	0.84	5.6 × 10^11^ ± 2.8 × 10^10^

nm: nanometer, PdI: polydispersity index, ZP: zeta potential in millivolts (mV), UroGm: Uroguanylin derivative.

**Figure 3:**
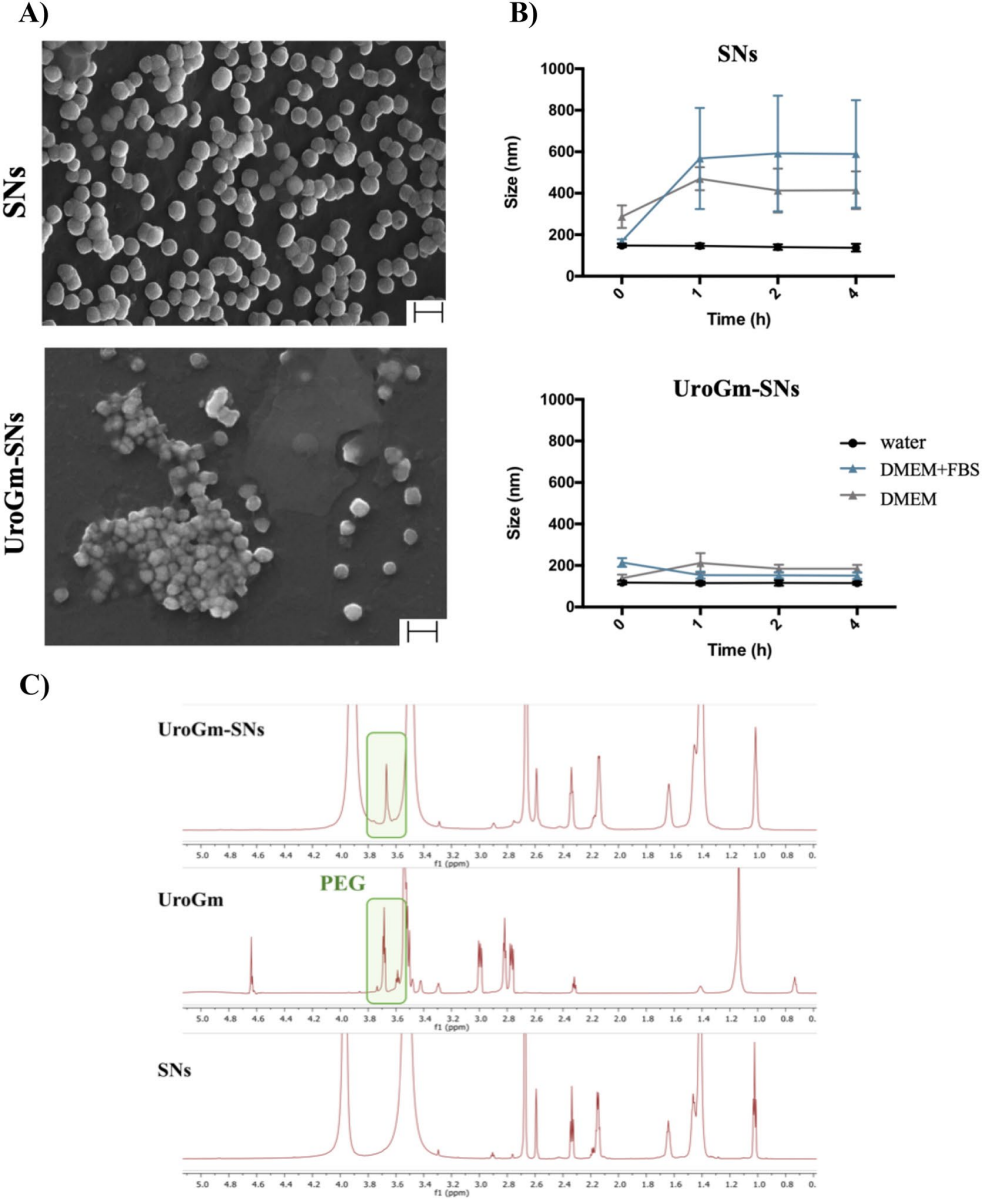
(**A**) Scanning transmission electron microscope images of SNs and UroGm-decorated SNs using InLens (immersion lens) mode. Scale bar = 200 nm. (**B**) Colloidal stability of non-decorated (SNs) and decorated (UroGm-SNs) nanosystems assessed at 37 °C in different media (supplemented or not with FBS). DMEM: Dulbecco's Modified Eagle Medium; FBS: Fetal Bovine Serum. (**C**) Comparison between the ^1^H-NMR spectrum of functionalized nanosystems (UroG-SNs), UroG-PEG_12_-C_18_ spectrum and the sphingomyelin nanosystems (SNs).

Particle concentration measurements established an average of 5 × 10^11^ particles/mL, irrespective of the presence of peptide. Morphological examination was subsequently performed by Field Emission Scanning Electron Microscopy (FESEM). The images in Fig. 3A showed a defined spherical shape that corroborated the same size values determined by previous characterization techniques, thus highlighting the robustness of the system. Interestingly, the morphology of the UroGm-SNs was found to be more irregular than non-decorated SNs, which may be due to the presence of a more susceptible to hydration part (corresponding with the PEG chain and UroG moieties) decorating the surface of the nanosystems (Fig. 3B). Stability of SNs and UroGm-SNs in different conditions (culture medium with and without FBS) was next assessed at 37 °C for 4 h. The results show a good colloidal stability in suspension (water) for both nanosystems (SNs and UroGm-SNs). Nevertheless, significant differences were found when incubated with supplemented and non-supplemented cell culture medium (DMEM). SNs suffered a major increase in size during the first hour of incubation in these media, thus suggesting a strong interaction with serum proteins (FBS). However, the size of the decorated nanosystems (UroGm-SNs) remained constant at the tested conditions, indicating an improved stability due to the presence of the amphiphilic UroGm at the interface. This fact can be related to the presence of the lipid-PEG derivative, since PEG chains are well known to avoid aggregation, stabilize the particles in cellular medium by steric hydration repulsions and prevent the adsorption of proteins^46^.

Additional evidence of the incorporation of UroGm to SNs was obtained by NMR analysis. Figure 3C shows the appearance of a signal from the PEG_12_ peak (δ 3.69–3.71) in the spectra of the UroGm, which is also observed in spectra of the functionalized UroGm-SNs, but not in that of non-functionalized SNs. For an accurate integration of UroGm, NMR signals corresponding to PEG_12_ peak were normalized to an internal control (TSP).

Although many reports in the literature have shown diverse ways to conjugate ligands to the nanosystems surface, determination of ligand density is rarely specified. Calculation of the precise amount of UroGm was done as well by NMR, revealing an actual concentration of UroGm in the formulation of 2.08 ± 0.14 µg/mL. Following Eqs. (1a) and (1b) (as detailed in methods section), we estimated a density of 0.012 UroGm molecules/nm^2^. These values are in line with other works reporting values from 0.225 to 0.005 molecules/nm^2^^34,47^.

**Figure 4:**
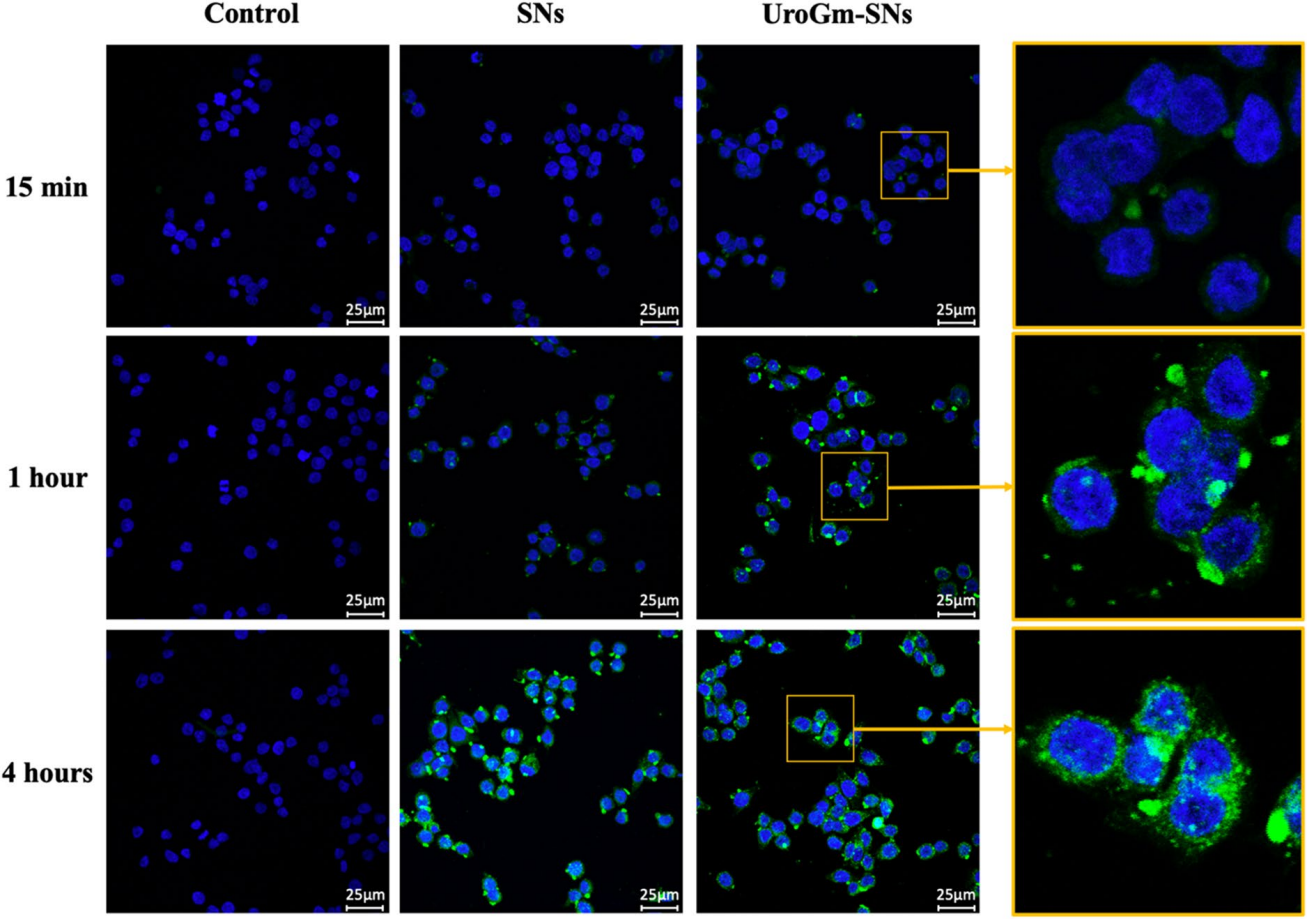
Confocal microscope images showing the internalization of SNs and UroGm-SNs in SW620 cells after 15 min, 1 h and 4 h of incubation. Green channel: TopFluor-sphingomyelin (TopFluor-SM) labelled nanosystems. Blue channel: nuclei stained with Hoechst.

Next studies were intended to get a first insight of the therapeutic potential of this formulation for treating colorectal cancer. Firstly, the capacity of UroGm-SNs fluorescently labeled with TopFluor-sphingomyelin to improve the interaction of SNs with colorectal cancer cells was studied by confocal microscopy in a metastatic colorectal cancer cell line constitutively expressing the GCC receptor, SW620 (Figure S3). Results showed that, after 1 h of incubation, the green fluorescence intensity, associated to the formulation, was higher for cells incubated with UroGm-SNs with respect to plain SNs (Fig. 4). Improved internalization capacity at shorter incubation times, being indicative of a specific interaction with the receptor of interest, has been previously reported for other targeted nanosystems decorated with transferrin^48^, folate^49^ and antiangiogenic peptides^50^. Although additional experiments would be needed to perform a deep comprehension of the interaction between UroGm-SNs and GCC receptor, we can state that our formulation shows a better interaction with the targeted cells and that UroGm is playing a positive role.

### Development of a combination nanotherapy

The use of combination therapies involving the incorporation of two anticancer agents into a nanosystem may provide benefits in the sense that different drugs may attack cancer cells at varying stages of their growth cycle^51^. Considering this, combination nanomedicine should be designed in such a way that it targets multiple signaling pathways with limited toxicity^30^. After proving the efficient association of UroGm to SNs and the capacity of UroGm-SNs to efficiently accumulate in metastatic colorectal cancer cells, we proceed with the encapsulation of the anticancer drug etoposide, a Topoisomerase II inhibitor described to be an alternative drug for treating advanced colorectal cancer^52–54^. As observed in Table 2, encapsulation of Etp did not alter the physicochemical properties of SNs and UroGm-SNs. Etp-loaded formulations maintained a small size, below 150 nm, a homogeneous particle distribution, and a negative zeta potential. The amount of etoposide loaded into the UroGm-Etp-SNs was determined by HPLC (40.51 ± 5 µg/mL). Importantly, the encapsulation of Etp did not interfere with the association efficiency of UroGm (RMN analysis showing similar peptide concentration (2.30 ± 0.12 vs. 2.08 ± 0.14 µg/mL) in UroGm-Etp-SNs and UroGm-SNs, respectively.

**Table 2:** Physicochemical characterization of UroGm-Etp-SNs.

Formulation	Zetasizer			Nanosight					
	Size (nm)	PdI	ZP (mV)	Size (nm)	D_10_	D_50_	D_90_	SPAN	Conc. (particles/mL)
Etp-SNs	141 ± 8	0.2	− 34 ± 3	155 ± 3	110 ± 2	141 ± 3	209 ± 9	0.70	4.3 × 10^11^ ± 3.3 × 10^10^
UroGm-Etp-SNs	126 ± 14	0.2	− 45 ± 5	122 ± 2	81 ± 1	108 ± 2	173 ± 3	0.85	5.6 × 10^11^ ± 1.8 × 10^10^

nm: nanometer, PdI: polydispersity index, ZP: zeta potential in millivolts (mV), SNs: Sphingomyelin nanosystems, Etp: Etoposide, UroGm: Modified Uroguanylin, (mean ± SD; n ≥ 3).

**Figure 5:**
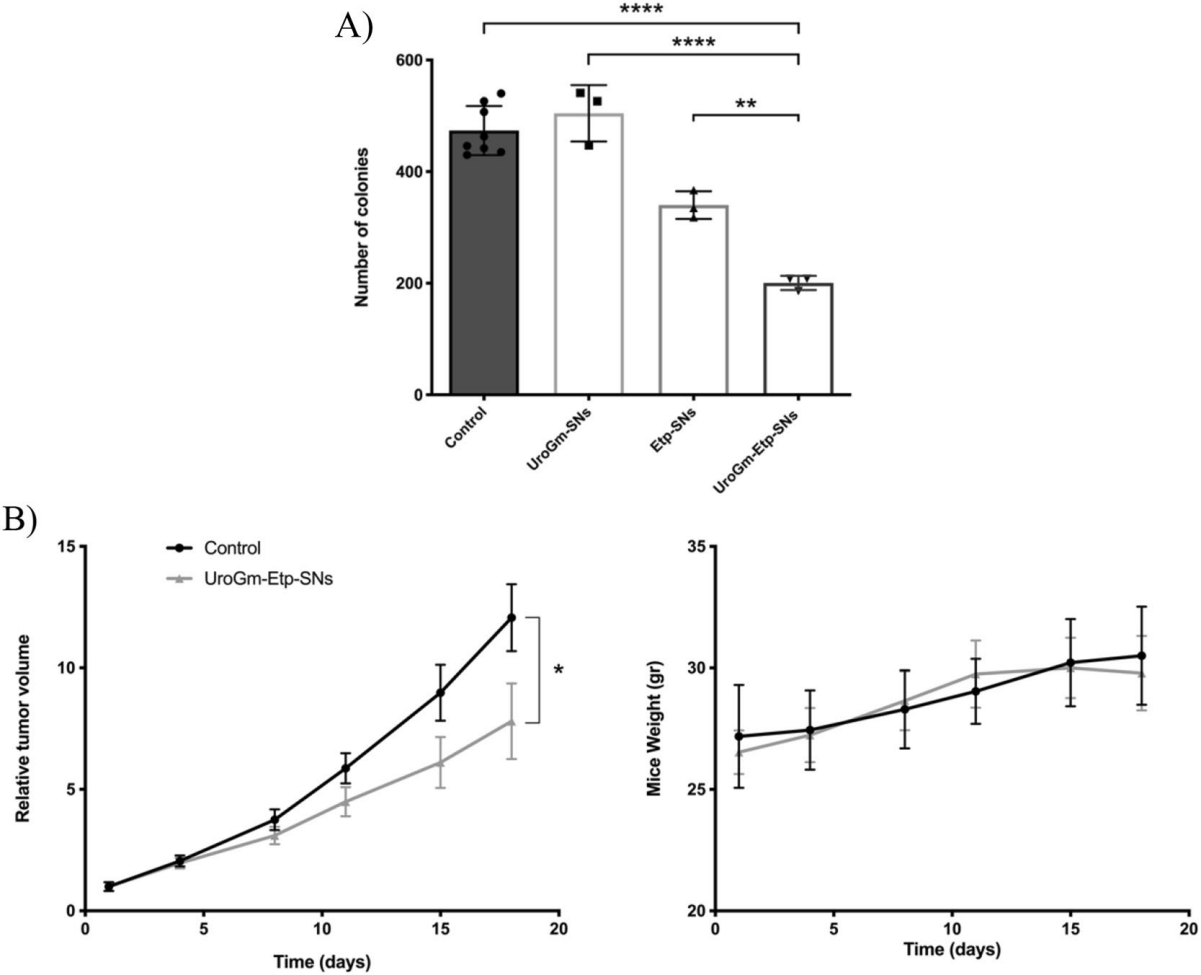
Graphical representation of (**A**) in vitro treatments evaluated in this work (UroGm-SNs, Etp-SNs and the combination UroGm-Etp-SNs) all at the same concentration of the active drugs (500 nM Etp and 50 nM UroGm). (**B**) In vivo* a*ntitumor effect in terms of relative tumor volume growth and evaluation of mice body weight. Data presented for control mice and UroGm-Etp-SNs treated mice (n = 6). *P* values: ****p < 0.0001, **p between 0.001 and 0.01 and *p < 0.05.

Taking advantage from the dual function of the UroGm (targeting and therapy) and having encapsulated Etp, the potential of this combination therapy was assessed in vitro in a metastatic colorectal cancer cell line. Preliminary CFA studies with the molecules in solution weresolutions of all single reagents performed. We observed that by combining separated drugs at subtherapeutic concentrations (as previously determined, ≤ 500 nM of etoposide and ≤ 50 nM of UroGm, Figure S4 and Fig. 2, respectively), a potentiated effect was achieved (Figure S5). As shown in Fig. 5A, a concentration of 50 nM/500 nM of UroGm/Etp loaded into SNs (UroGm-Etp-SNs) can significantly reduce colony formation, with respect to the control, and also to the reference formulations loaded with each drug separately (UroGm-SNs and Etp-SNs). This could be justified due to the complementary mechanism of action of both molecules (i.e. activation of cGMP-AKT axis by UroGm and DNA damage triggered by Etp^55,56^), and to an improved uptake of Etp into GCC expressing cells due to its encapsulation into the targeted nanosystem, as disclosed for other works^57,58^.

Finally, in vivo results confirmed the activity of the proposed therapy, UroGm-Etp-SNs, comparing the tumor growth of mice treated with this formulation with a control group. Importantly, given the limitations of the formulation, as the synthesis of UroGm should be scaled-up prior performing more comprehensive studies, compositions that proved not to be as efficient in vitro (UroGm-SNs and Etp-SNs), were not included in the study. The results of the relative tumor volume represented in Fig. 5B, encouragingly show a significant but modest reduction of the tumor growth with time. No alteration was observed in the mice weight. Importantly, the administered dose of etoposide was significantly lower than that reported for other type of etoposide-loaded nanosystems for the treatment of melanoma (2 mg/kg vs. 50 mg/kg)^58^. Dose-schedule optimization could lead to improved outcomes, studies that will be the subject of future work. Additionally, these results are encouraging and can trigger further studies in mice bearing orthotropic tumors and in models of liver metastatic disease to fully disclose the potential of UroG-decorated nanosystems for targeted drug delivery to colorectal cancer.

## Conclusion

In this work we have shown the potential of SNs to develop targeted combination treatments for metastatic colorectal cancer. We selected uroguanylin (UroG) as a dual-acting molecule, acting as ligand on the nanoparticle surface, while simultaneously inducing a therapeutic response by activating GCC receptor. We successfully developed an amphiphilic derivative of uroguanylin (UroGm) to facilitate its anchoring onto the nanoparticle surface without losing the therapeutic properties of the native molecule. Encapsulation of etoposide (Etp), a second anticancer drug, was addressed next. The resulting nanosystems UroGm-Etp-SNs was extensively evaluated in vitro and preliminary tested in mice bearing xenograft SW620 cells. Our results show that delivering both drugs in combination provided a remarkable therapeutic response, opening up novel strategic venues for treating metastatic colorectal cancer.

## Methods

### Materials

C_18_-PEG_12_-COOH (MW 825 g/mol) was obtained from Creative PEGWorks (Winston Salem, NC, USA). 4-(4,6-Dimethoxy[1,3,5]triazin-2-yl)-4-methylmorpholinium chloride salt (DMTMM·Cl, MW 276.72 g/mol) was purchased from Sigma Aldrich (Madrid, Spain). Uroguanylin (UroG, MW 1667.9 Da; NDDCELCVNACTGCL) was purchased from Bachem (King of Prusia, PA, USA). Oleic Acid was acquired from Sigma Aldrich (Madrid, Spain). Sphingomyelin (Lipoid E SM) was kindly provided by Lipoid GmbH (Ludwigshafen, Germany). Etoposide (purity ≥ 98%) was purchased from Cayman Chemical Company (Ann Arbor, MI, USA). MiniDyalisis Kit, 1 kDa cut-off was obtained from GE Healthcare (GE Healthcare Bio-Science Corp., NJ, USA). HPLC grade Acetonitrile (ACN) and Ethanol (EtOH) were obtained from Fisher Chemicals (Thermo Fisher Scientific, USA) and Trifluoroacetic acid (TFA) was provided by Sigma-Aldrich (Madrid, Spain). Dimethyl sulfoxide (DMSO, 99.8% D) was purchased from (Cortecnet Inc., Paris, France). All other chemicals used were HPLC or UPLC purity grade.

### Synthesis and characterization of uroguanylin derivative (UroGm)

Uroguanylin (UroG) was covalently linked to C_18_-PEG_12_-COOH through an amide linker. As carboxyl activating agent, DMTMM was used^56^. Firstly, stock solutions of all single reagents were prepared: C_18_-PEG_12_-COOH and DMTMM were dissolved at 40 mg/mL in MilliQ water and UroG was dissolved at 1 mg/mL in HEPES 300 mM buffer (pH = 8)^15^. DMTMM (120 eq, 276.72 g/mol) was added over C_18_-PEG_12_-COOH solution (100 eq, 825 g/mol) under magnetic stirring and left for 10 min at room temperature (RT) to promote the activation of the carboxylic groups. Then, 200µL of UroG stock solution (1 eq, 1667, 9 Da) were added dropwise to the previous mixture. HEPES buffer was used to adjust the pH to 7.6 with a final buffer concentration of 150 mM. The reaction was allowed to proceed for 8 h at RT. For purification, the reaction volume was dialyzed against deionized water 3 times for 20 h by using a MiniDyalisis Kit (MWCO 1 kDa) and then analyzed by HPLC, NMR and MALDI-TOF techniques (detailed information in supplementary materials).

### Preparation of UroGm-SNs

Sphingomyelin nanosystems incorporating the modified Uroguanylin (UroGm) were prepared by ethanol injection technique. Briefly, UroGm was dissolved in water at a concentration of 0.5 mg/mL. On the other hand, oleic acid and sphingomyelin were dissolved in ethanol at a concentration of 200 mg/mL and 40 mg/mL respectively. Subsequently, 50 μL of the oily phase (composed by 2.5 mg of oil and 0.5 mg of surfactant) were injected into 450 µL of ultrapure water (containing the appropriate quantity of UroGm) under continuous magnetic stirring and nanosystems were spontaneously formed. Increasing amounts of UroGm were added to the formulation in order to explore the maximum loading capacity (data not shown) establishing a final amount of 10 µg of UroGm per formulation as the best condition. Formulations were then isolated by centrifugation (20,000 RFC for 45 min at 15 °C) using an Eppendorf 5417R centrifuge (Eppendorf, Germany) to purify the nanosystems.

### Physicochemical characterization

Particle size and polydispersity index (PdI) were determined by Dynamic Light Scattering (DLS), and Z-potential values by Laser Doppler Anemometry (LDA), using a Zetasizer NanoZS (Malvern Instruments, UK). Measurements were performed at 25 °C with a detection angle of 173º upon 1/10 dilution with ultrapure water (MilliQ). Nanosystems were additionally characterized by nanoparticle tracking analysis (NTA), a method to measure particle size based on imaging of individual nanosystems. Experiments were conducted with a NanoSight NS3000 System (laser operating at λ = 488 nm) (Malvern Instruments, UK). Briefly, nanosystems were injected in the sample chamber at a 1000-fold dilution in ultrapure water. Five captures, with a camera level of 14, were used to determine several parameters such as average size, homogeneity and particle concentration. Colloidal stability of the nanosystems was determined after being stored at 4 and 37 °C, as well as after incubation in biological media (DMEM high glucose, Sigma Aldrich) supplemented or not with 1% v/v fetal bovine serum (FBS, Gibco).

### Morphological examination

Morphological examination of the formulation was performed by Field Emission Scanning Electron Microscopy (FESEM) Ultra Plus (Zeiss, Germany) configured with InLens and STEM modes and operating at 20 kV. For the preparation of FESEM samples, 20 µL of the nanosystem suspension were mixed with 20 µL of 2% (w/v) phosphotungstic acid and stained for 6 h. The mixture was placed onto a copper grid with a formvar-carbon film, washed with 500 µL of ultrapure water and dried overnight in a desiccator under vacuum.

### Ligand density calculation

Efficient incorporation of UroGm into the nanosystems surface was determined by NMR. To achieve an accurate quantification, a fraction of the non-isolated UroGm-SNs were collected to quantify the precise total amount of UroGm presented in the formulation. After isolating the nanosystems, both the supernatant (where the decorated UroGm-SNs are located) and the undernatant (containing the free compounds in solution) were collected for further analysis. These three fractions (i.e. total, supernatant and undernatant) were freeze dried to remove traces of ethanol that were found to interfere with the analysis (peaks of ethanol overlap with the peak of the PEG in the ^1^H-NMR spectrum), and eventually dissolved in 500 µL of deuterated DMSO (99.8% D). NMR experiments were conducted at 25 °C on a Bruker NEO 17.6 T spectrometer (proton resonance 750 MHz) (Bruker, US), equipped with a ^1^H/^13^C/^15^N triple resonance probe and shielded PFG z-gradient. All the spectra were processed with MestreNova software v12.0 (Mestrelab Research Inc*.*, Spain). The chemical shifts were referenced automatically with respect to the deuterium lock. Samples were prepared in 5 mm thin wall NMR tubes. A 1D proton spectra (^1^H) was acquired for each sample using the pulse-acquisition sequence. The spectrum was acquired under quantitative conditions by using a low excitation tilt pulse angle of only 30°, an inter-scan delay (d_1_) of 6 s and an acquisition time (*aq*) of 2.75 s. The proton spectrum was processed with Fourier transformation and the phase and baseline were carefully corrected. For control, plain SNs were also prepared and characterized following the same methodologies, without the addition of UroGm in the aqueous phase. Surface density of UroGm molecules was subsequently calculated as the number of molecules per surface unit of nanosystem (nm^2^). Firstly, the number of UroGm particles were calculated with Eqs. (1a) from the previously NMR determined concentration. On the other side, nanosystems surface area were calculated using Eq. (1b), considering SNs morphology as perfect spheres, and with the concentration and radium parameters obtained from NTA measurements.

Equation 1: Formulas for calculation of number of particles (a) and surface area of a sphere (b).

1a $$ N_{UroGm} = \frac{{Mass \times N_{A} }}{MW} $$

1b $$ SA_{SNs} = 4\pi r^{2} $$

N_UroGm_: number of UroGm molecules; NA: Avogadro Constant; MW: molecular weight; SA_SNs_: SNs Surface Area; r^2^: radius squared.

### Preparation of dual-loaded SNs

UroGm-functionalized sphingomyelin nanosystems (UroGm-SNs) were additionally loaded with the chemotherapeutic drug etoposide (UroGm-Etp-SNs). In this case, up to 250 µg of etoposide (40 mg/mL in DMSO) were placed into the organic phase within the 50 µL of ethanol and injected into the 450 µL of ultrapure water containing UroGm. Nanosystems were isolated using the same conditions as previously described. Encapsulation efficiency was determined by direct quantification of etoposide in the nanosystem using an isocratic HPLC method optimized from the literature^57^. Analyses were performed in an HPLC system 1260 Infinity II Agilent (Agilent Technologies, USA) equipped with a pump G7111A, an autosampler G7129A and an UV–Vis detector G7114A set at 254 nm. Separation was achieved on an InfinityLab Poroshell 120 EC-C18 (100 mm × 4.6 mm, 4 µm pore size) Agilent column. The mobile phases were composed of water and acetonitrile (H_2_O:ACN,70:30 v/v) at a flow rate of 1 mL/min. Standard calibration curves were linear in the range of 1 to 15 µg/mL (R^2^ = 0.9999) (Limit of quantification, LOQ = 1 ppm).

### Cell viability studies

Cell toxicity analyses were performed to determine the viability of metastatic colorectal cancer cells SW620 (ATCC CCL-227) upon exposure to increasing concentrations of SNs (from 0.01 to 10 mg/mL) in a final volume of 150 µL (25 µL corresponding to the nanosystem and 125 µL to complete medium). Etp-SNs were also tested to evaluate the effect of encapsulating the cytostatic drug. Cells were seeded at a density of 10.000 cells/well in 96-well plates 24 h before the experiment. After 48 h of incubation with SNs and Etp-SNs, medium was removed and 100 µL of tetrazolium dye (3-(4,5-dimethylthiazol-2-yl)-2,5-diphenyltetrazolium bromide, MTT) solution (5 mg/mL in PBS, MTT Alfa Aesar, Germany) were added to each well. After 3 h of incubation this solution was also removed, and formazan crystals were solubilized with 100 µL of DMSO and maintained at 37 °C for 15 min protected from light. Results were obtained by measuring absorbance at 570 nm in a microplate spectrophotometer (Multiskan EX, Thermo Labsystems). Cell viability in percentage (%) was calculated in comparison with control wells containing untreated cells.

### Cellular internalization studies

Internalization studies in SW620 metastatic cancer cells were performed by confocal microscopy (Leica SP8, Germany). Fluorescent UroGm-SNs were prepared by adding the modified lipid TopFluor-sphingomyelin in their composition (0.5 µg/nanosystem). To evaluate cellular uptake, 200,000 cells were seeded on a 24-well plate over a glass coverslip. After 24 h, the cells were washed with PBS and then incubated for up to 4 h with Etp-SNs, UroGm-SNs and UroGm-Etp-SNs at a concentration of 0.13 mg/mL per well (added onto 500 µL of cell culture medium). After this period, the medium was removed and cells were washed twice with PBS. Then, they were fixed with paraformaldehyde (4% w/v) for 15 min and then washed with PBS. Cellular nuclei were stained with Hoechst 33342 (Invitrogen, US) for 5 min and then cells were washed three times with PBS. Finally, the coverslips were mounted over microscope slides using 8 µL of Mowiol mounting medium (Calbiochem, USA). Coverslips were dried in the dark overnight (at room temperature) before visualization.

### Colony forming assays

SW620 colorectal cancer cells were plated in triplicate at a density of 600 cells/well in 12-well plates and cultured in a humidified incubator (37 °C, atmosphere of 5% CO_2_ and 95% RH). Drug treatments were maintained in contact with cells for the complete duration of the experiment (15 days). After this period cells were stained with an MTT solution (5 mg/mL) for 3–4 h and subsequently dried and scanned. Obtained images were analyzed using ImageJ software (version 1.53k, Wayne Rasband NIH, USA). In vitro differences were statistically determined by one-way ANOVA (GraphPad PRISM, version 6.0, GraphPad Software, Inc., USA).

### In vivo efficacy of UroGm-Etp-SNs in mice bearing SW620 xenografts

In order to perform the in vivo assays, 5 × 10^6^ SW620 colon cancer cells (ATCC CCL-227) dispersed in 100µL of growth media and Matrigel (BD Bioscences) (3:1) were injected in both flanks of female NMRI-nu mice 4–6 weeks old. Tumor growth was quantified by serial caliper measurements, body weights were recorded, and tumor volumes were calculated (V = πd3/6). UroGm-Etp-SNs (with a UroGm dose 0.05 mg/kg and Etp 0.5 mg/kg) were administered at days 4, 7, 11 and 15 of the study (Figure S6). In vivo differences were statistically determined by one-way ANOVA (GraphPad PRISM, version 6.0, GraphPad Software, Inc.). Statistical analysis was performed with multiple Student’s t test. When differences were detected from t test, Wilcoxon signed rank test was used for pairwise differences between control and treatment groups.

In vivo studies were performed at the animal facility of CiMUS, Santiago de Compostela (register no. 150780275701) by the group of Dr. Anxo Vidal (University of Santiago de Compostela). All animal procedures were approved by the competent authority (Xunta de Galicia, authorization ID: 15010/14/001, “Avaliación biolóxica de nanosistemas”) after a positive report of the Bioethics Committee of the University de Santiago de Compostela. All experiments were executed in compliance with the Directive 2010/63/EU of the European Parliament and Council of 22nd September 2010 on the protection of animals used for scientific purposes, with the ARRIVE guidelines and with the Spanish Royal Decree 53/2013 February 1st on the protection of animals used for experimental and other scientific purposes, and following the Three R’s principle of animal research.

## Supplementary Information

Supplementary Information.

## Data Availability

The datasets used and/or analyzed during the current study are available from the corresponding author on reasonable request.

## Abbreviations

SNs: Sphingomyelin nanosystems
UroG: Uroguanylin
UroGm: Uroguanylin hydrophobic derivative
Etp: Etoposide
GCC: Guanylyl cyclase C receptor
PEG: Poly(ethylene glycol)
CFA: Colony forming assay
