# Supplementary material for: Sphingomyelin nanosystems loaded with uroguanylin and etoposide for treating metastatic colorectal cancer
Source: Sci Rep. 2021 Aug 26;11:17213. doi: 10.1038/s41598-021-96578-z (PMC8390746; doi:10.1038/s41598-021-96578-z)
Supplement: Supplementary file 1 — Supplementary Information. [file 41598_2021_96578_MOESM1_ESM.docx]

**Sphingomyelin nanosystems loaded with uroguanylin and etoposide for treating metastatic colorectal cancer**

*Belén L. Bouzo^a,b^*^‡^*, Sainza Lores^a^*^‡^*, Raneem Jatal^a^, Sandra Alijas^a^, María José Alonso^b,c,d^, Inmaculada Conejos-Sánchez^b^, María de la Fuente^a*^*

^a^ Nano-Oncology and Translational Therapeutics Unit, Health Research Institute of Santiago de Compostela (IDIS), SERGAS, CIBERONC, 15706 Santiago de Compostela, Spain.

^b^ Centre for Research in Molecular Medicine and Chronic Diseases (CIMUS), Av. Barcelona s/n Campus Vida. University of Santiago de Compostela (USC) 15706 Santiago de Compostela, Spain

^c^ Health Research Institute of Santiago de Compostela (IDIS), 15706 Santiago de Compostela, Spain.

^d^ Faculty of Pharmacy, University of Santiago de Compostela, Spain

**SUPPORTING INFORMATION**

**METHODS**

***NMR***

NMR experiments were conducted at 25˚C on a Varian Inova 17.6 T spectrometer (proton resonance 750 MHz) (Agilent Technologies, Inc., US), equipped with a 1H/13C/31P triple resonance probe and shielded PFG gradients. The spectrometer control software was VNMRJ v3.2 (Agilent Technologies, Inc., US). All the spectra were processed with MestreNova software v11.0 (Mestrelab Research Inc., Spain). The chemical shifts were referenced automatically with respect to the deuterium lock. Samples were prepared in standard 5 mm NMR tubes by dissolving 5 to 10 mg of the product in 50 µL of D_2_O and 550 µL of H_2_O. 1D proton spectra (^1^H) were acquired for each sample using strong suppression of the water peak with the WET scheme [1]. Each spectrum was measured with 64 scans with an inter-scan delay (d1) of 1.4 s and the acquisition time (at) was 0.4 s. DOSY spectra were performed with the sequence Doneshot [2]. This sequence is based in the Bipolar Pulse STimulated Echo and was modified to incorporate a watergate 3-9-19 scheme [3] for the suppression of the strong H_2_O solvent peak at ~ 4.71 ppm. Each PFG gradient had a rectangular shape and after its application it was followed by stabilization delay 0.4 ms. The pair of bipolar PFG gradients for encoding/decoding diffusion had a total duration (δ) of 3 ms. The diffusion delay (Δ) was set to 300 ms. The diffusion dimension was obtained by linearly varying the strength of the bipolar gradients in 32 steps from 2.5 to 50 G cm^-1^. Sixteen scans were measured per each point in the diffusion dimension. The intensity of each peak in the 1D sub-spectrum was fitted to the Stejskal-Tanner equation to determine the diffusion coefficients of the signals [4] by using the analysis module of MestreNova software.

A 2D TOCSY spectrum was measured for UroG-PEG_12_-C_18_ (UroGm) and UroG samples with a TOCSY sequence that includes a flipback pulse and watergate scheme for the suppression of the strong water signal [3]. The TOCSY period was based on a DIPSI-2 scheme and was applied with a field strength of 8.3 kHz during a mixing time of 80 ms. The spectrum was registered with 48 scans per t1 increment and the number of complex points registered were 4096 and 180 for the T2 and T1 dimensions.

***MALDI-TOF***

Mass spectra analyses were carried out in an ULTRAFLEX III MALDI TOF/TOF mass spectrometer (Bruker Daltonics, Germany) equipped with a 200 Hz smartbeam laser. Samples were spotted onto a HCCA matrix (α-Cyano-4-hydroxycinnamic acid). Calibration of the mass spectra was performed using a standard peptide mixture. MS spectra were acquired in the reflectron positive ion mode within a mass range from 700 to 2000 Da and 25 kV settled voltage.

***HPLC***

HPLC analyses were performed in a HPLC System LaChrom Elite, VWR-Hitachi, equipped with an autosampler L-2200, pump model L-2130, oven L-2300 and a UV-detector L-2400 settled at 220nm wavelength (Hitachi High Technologies America, Inc., US). A Kinetex C8 column (5µm, 100Å pore size, 4.6 mm × 150 mm) (Phenomenex Inc., US) operating at 40 ºC was used for separation. The mobile phases used were: Solvent A (water 98%, ACN 2%, 0.1% TFA) and Solvent B (ACN 95%, water 5%, 0.1% TFA). Flow rate was set at 0.6 mL/min. Separation was achieved using the following mobile phase gradient: t= 0, 0% B; t=1 min, 0% B; t= 2min, 10% B; t= 5 min, 23% B; t= 9 min, 34% B; t= 11 min, 80% B; t= 12min, 0% B and t= 15 min, 0% B.

***GCC expression in colorectal cancer cells***

Two colorectal cancer cell lines were evaluated regarding the expression of GCC for comparison purposes. HT29 a cell line isolated from a primary tumor that mimics many of the properties of the mature intestine (ATCC HTB-38, cultured in McCoy 5A medium (Sigma Aldrich, Spain)) and SW620, a metastatic cell line isolated from a lymph node from a primary adenocarcinoma (ATCC CCL-227, cultured in Dulbecco’s Modified Eagle’s Medium (DMEM) High glucose (Sigma Aldrich, Spain)). Both media were supplemented with 10% (v/v) Fetal Bovine Serum (FBS) (Gibco, Life Technologies, US) and 1% (v/v) penicillin/streptomycin (Sigma Aldrich, Spain) and cells were maintained at 37ºC in 5% CO_2_ atmosphere. To analyze the expression of the GCC receptor, 100.000 to 150.000 cells were seeded in an 8 wells µ-chamber slides (PCL30108, SPL LifeScience, Korea). After 24 h, cells were fixed using paraformaldehyde 4% (PFA 4%) at room temperature for 15 minutes, permeabilized for 10 minutes with Triton X-100 0.2% and blocked with BSA 3% in PBS for 1 hour. Then, the polyclonal antibody rabbit anti-human GCC antibody (ab213430, Abcam, UK) was incubated for 1 hour at a 1:1000 dilution (BSA 2% in PBS). Secondary antibody Alexa Fluor 488 polyclonal antibody goat anti-rabbit IgG (ab150077, Abcam, UK) was incubated for 1 h at room temperature in the dark (BSA 2% in PBS). Cell nuclei were stained with Hoechst 33342 (Invitrogen, US) (1:1000 dilution in PBS). Finally, cells were visualized under the confocal microscope (Leica SP8, Germany) (**Figure S3**). Cells counter-stained only with Hoechst and cells incubated only with the secondary antibody, were respectively used as control and negative staining for green secondary antibody signal.

**REFERENCES**

1 Smallcombe SH, Patt SL, Keifer PA. WET Solvent Suppression and Its Applications to LC NMR and High-Resolution NMR Spectroscopy. *Journal of Magnetic Resonance, Series A* 117(2), 295–303 (1995).

2 Pelta MD, Morris GA, Stchedroff MJ, Hammond SJ. A one-shot sequence for high-resolution diffusion-ordered spectroscopy. *Magnetic Resonance in Chemistry* 40(SPEC. ISS.), 147–152 (2002).

3 Liu M, Mao XA, Ye C, Huang H, Nicholson JK, Lindon JC. Improved Watergate Pulse Sequences for Solvent Suppression in NMR Spectroscopy. *Journal of Magnetic Resonance* 132(1), 125–129 (1998).

4 Koay CG, Özarslan E. Conceptual foundations of diffusion in magnetic resonance. *Concepts in Magnetic Resonance Part A: Bridging Education and Research* 42(4), 116–129 (2013).

**FIGURES**

**A)**

**
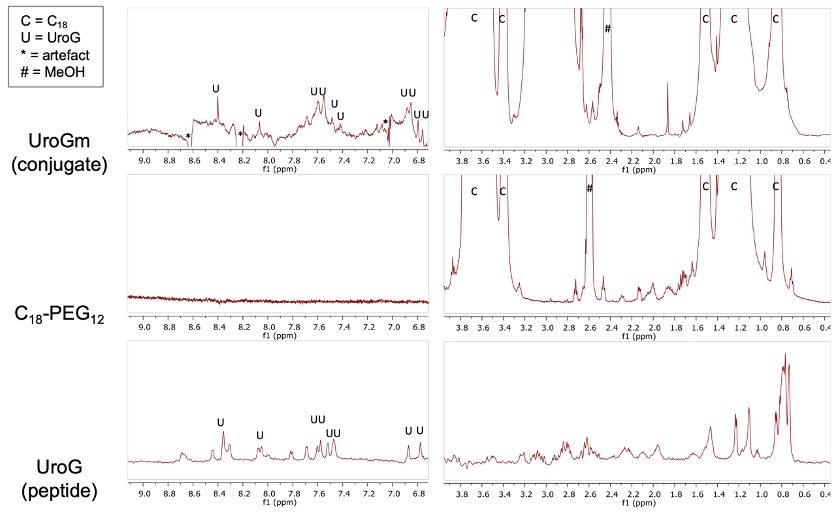
**

**B)**

**
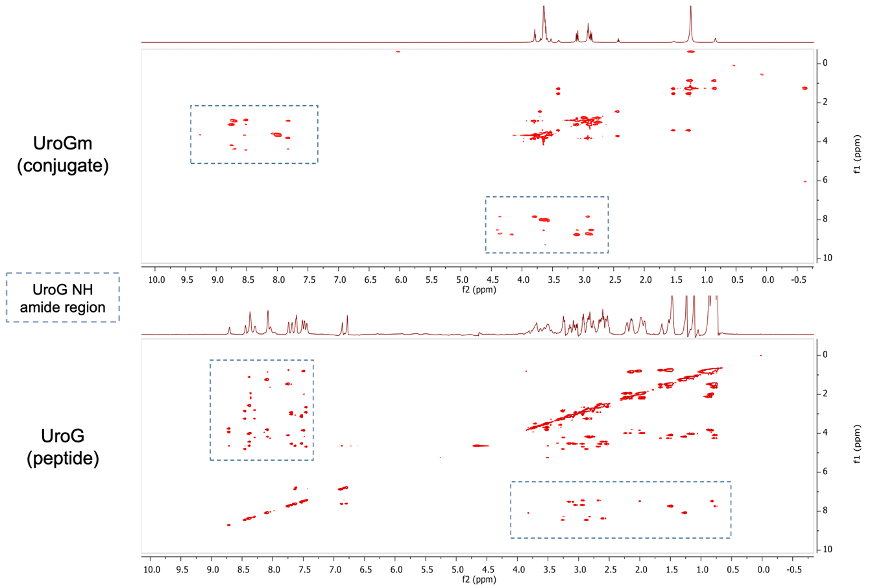
**

**Figure S1**. **(A)** ^1^H-NMR analysis of the reagents and product of the chemical conjugation. **(B)** TOCSY analysis of the amide region. *Spectra represented with MestreNova software v11.0 (Mestrelab Research Inc., Spain).*


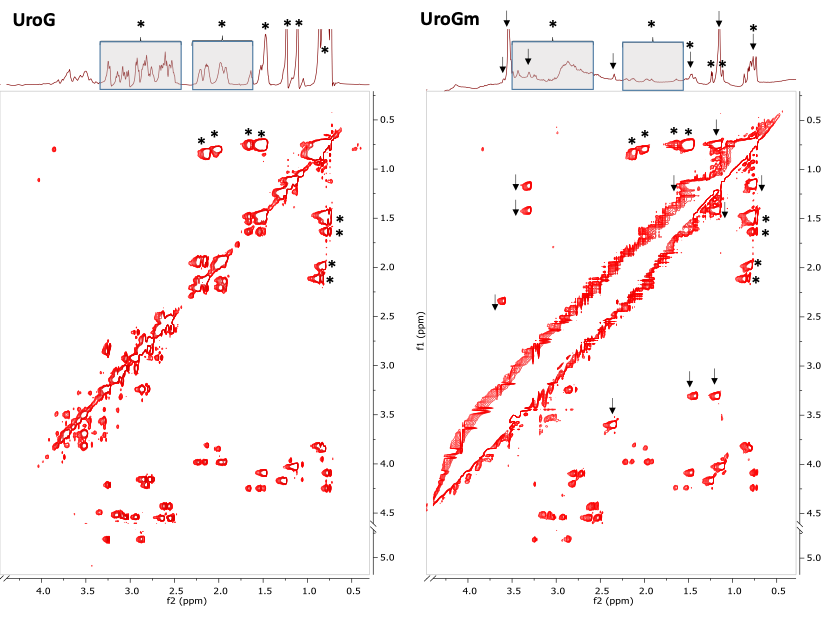


**Figure S2**. TOCSY analysis of the parent peptide UroG (left) and its conjugate UroGm (right) (*** = *UroG signals*, ↓*=PEG_12_-C_18_ signals*). *Spectra represented with MestreNova software v11.0 (Mestrelab Research Inc., Spain).*


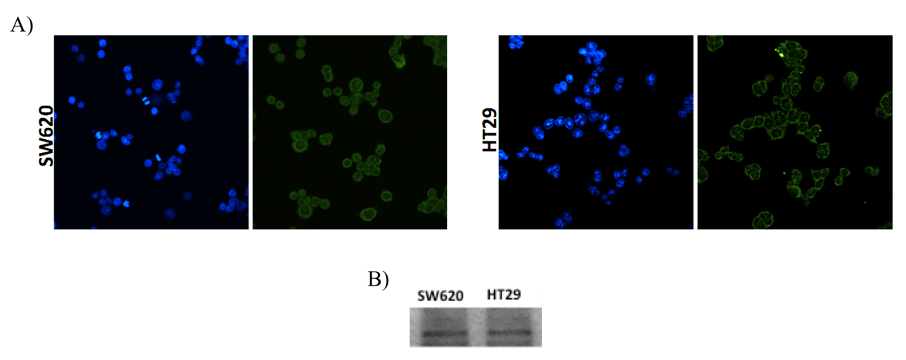


**Figure S3.** Immunohistochemistry images of GCC receptor in SW620 and HT29 colon cancer cell lines


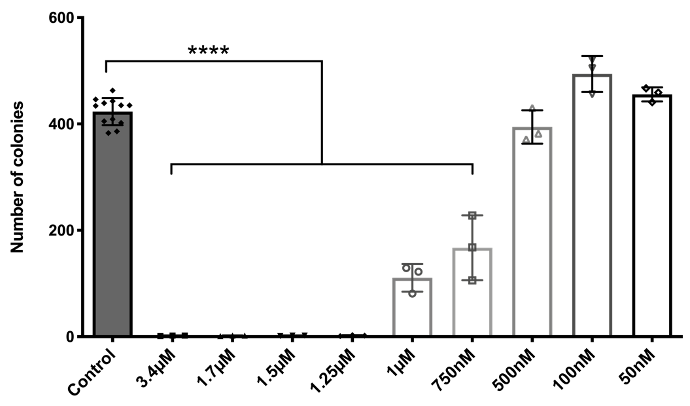


**Figure S4.** Evaluation of cell proliferation activity by colony forming assay (CFA) of SW620 cells treated with increasing amounts of the anticancer drug etoposide*,* P value **** p < 0.0001


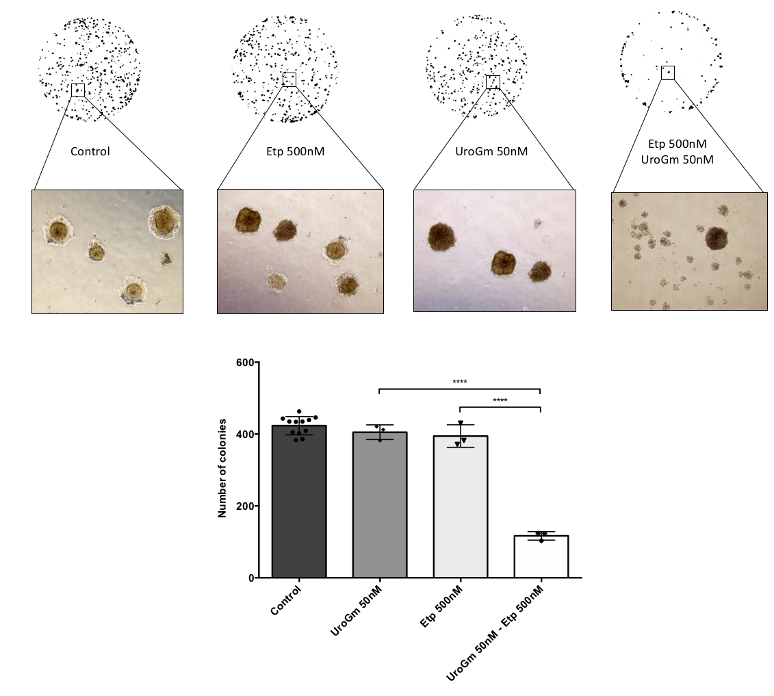


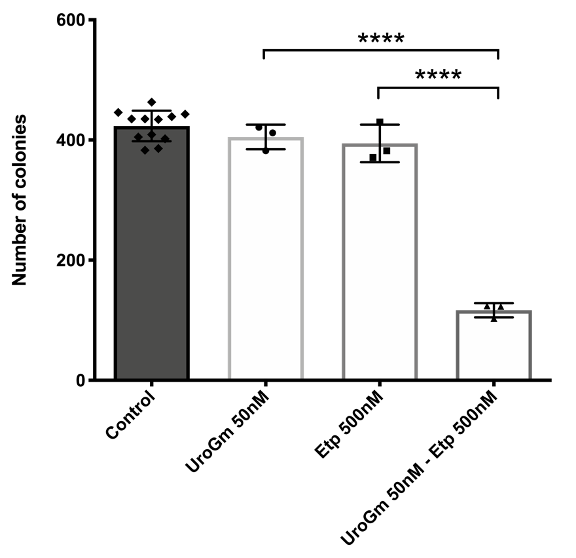


**Figure S5.** Evaluation of cell proliferation activity by colony forming assay (CFA). **(A)** Image of macroscopic colonies photographed from P12 well plates and cellular detail of each condition. **(B)** Graphical representation of the clear combination effect between UroGm and Etp in contrast with the toxicity exhibited by the isolated drugs. *P value **** p < 0.0001.*

**
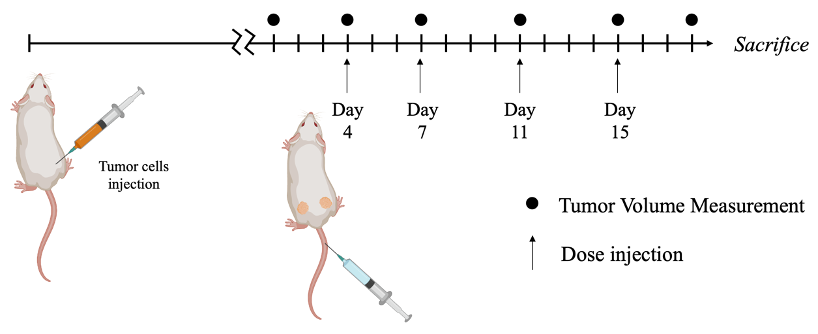
**

**Figure S6.** Experimental *in vivo* timeline schedule of multiple doses.
